# Facile Synthesis of N, S-Doped Carbon Quantum Dots from Food Waste as Fluorescent Probe for Sensitive Detection of Thiamphenicol and Its Analogues in Real Food Samples along with an Application in Bioimaging

**DOI:** 10.3390/foods11162414

**Published:** 2022-08-11

**Authors:** Shujuan Chen, Wanlin Ouyang, Yiting Zhu, Li He, Likou Zou, Xiaolin Ao, Shuliang Liu, Yong Yang, Jianlong Li

**Affiliations:** 1 College of Food Science, Sichuan Agricultural University, Ya’an 625014, China; 2 Yi’yang Agricultural Products Quality Inspection and Testing Center, Yi’yang 413000, China; 3 College of Resources, Sichuan Agricultural University, Chengdu 611130, China

**Keywords:** food waste, N, S-CDs, rapid detection, thiamphenicol, bioimaging

## Abstract

Herein, N, S co-doped carbon quantum dots (N, S-CDs) with high absolute quantitative yield (Abs-QY) of 50.2% were produced by hydrothermal treatment of food residue crayfish shells. A new detection method of thiamphenicol (TAP) and its analogues was established by discovering the obvious fluorescence response between TAP and N, S-CDs, which achieved a wide linear range of 20–300 μg·L^−1^ with a detection limit (LOD) of 11.12 μg·L^−1^. This novel probe exhibited strong sensitivity and shows rapid response in complex food matrices (overall detection time is less than 45 min) mainly induced by static quenching. Spiked food sample recovery ranged from 97.3 to 99.34%. Further, the cell experiments of N, S-CDs were conducted, and the cell viability remained 91.76% under high concentration of N, S-CDs due to the environmentally friendly materials. The low cytotoxicity and good cytocompatibility make these N, S-CDs compatible for cell bioimaging and intracellular detection of TAP.

## 1. Introduction

Amphenicol antibiotics are a kind of broad-spectrum antibiotic drugs [1], used widely for the veterinary treatment of food-producing animals. Chloramphenicol (CAP) represents amphenicol antibiotics, but it was prohibited in the European Community Regulations 1430/94 notice in 1994 [2]. Thiamphenicol (TAP) is similar in structure to CAP but much more potent [3] and is a perfect substitute for CAP in treating diseases of poultry and animals [4]. Several studies have documented that TAP has a broad spectrum of action against bacterial infections of the gastrointestinal and respiratory tract, bacterial prostatitis, and sexually transmitted diseases. Given the excellent antibacterial properties and fungibility, TAP is an attractive option in many countries for treating animal diseases. However, the overuse of TAP in animal feedstuffs can cause the presence of its residues in animal-derived foods and induce antibacterial resistance [5]. Moreover, it shows hematological toxicity, which presents a danger to human health. Hence, the European Economic Community (EEC) [6] has defined the maximum residue limit (MRL) of TAP in milk and poultry muscle tissues as 50 µg·kg^−1^. Currently, the conventional detection methods for TAP involve enzyme-linked immunosorbent (ELISA) [7] assay, UV spectrophotometry [8], high-performance liquid chromatography (HPLC) [9], and high-performance liquid chromatography–mass spectrometry (LC-MS) [10]. Among these, ELISA can rapidly achieve trace detection and cope with large number of samples but is still prone to false positives or negatives. The UV spectrophotometry is simple in operation but has a poor detection limit and selectivity. The HPLC and LC-MS method has its advantages of good sensitivity and accuracy; nevertheless, it requires long analysis time, tedious pretreatment steps, and many organic solvents. Considering that, it is vital to establish a novel method for the highly effective determination of TAP residues in food samples.

Fluorescence sensing detection method has been widely used due to excellent optical performance and high sensitivity in recent years. Several studies have shown that TAP detection techniques by semiconductor metal-quantum dot can obtain acceptable linear range and detection [11,12]. However, semiconductor quantum dots are primarily composed of II–VI or III–V metal elements, which still have biosecurity threats. Surprisingly, carbon quantum dots (CDs) display excellent photostability, favorable biocompatibility, and low toxicity, which is an alternative to the traditional semiconductor-quantum dots gradually [13]. Up to now, many natural food products are easily available to replace the usual chemicals for preparing CDs. The efficient utilization of food waste with rich carbon sources is a more favorable alternative option, with advantages of being cost-effective and eco-friendly [13,14]. CDs synthesized from natural resources still pose significant challenges because of their low quantum yield and poor stability. One of the most effective strategies to regulate the photoluminescence properties and quantum yield of CDs is heteroatom doping. For instance, nitrogen- and sulfur-doped CDs (N, S-CDs) possess abundant nitrogen- and sulfur-containing surface functional groups, which may bring better features and broader application prospects for the fluorescent materials [15]. Thus far, there have been several studies on the synthesis of N, S-CDs for applications such as detection of metal ion in lake water and environmental water (Table S1) [16,17,18,19,20,21,22]. However, the N, S-CDs prepared in these studies required special analytical grade reagents, and the fluorescence quantum yield and cytotoxicity were not satisfactory. In addition, these researches lacked research on the stability of probes as well as more detailed discussion of the fluorescence quenching mechanism. Dong prepared CDs, N-CDs, and N, S-CDs with different dopants, and among them, N, S-CDs showed better fluorescence (FL), quantum yield (QY), and fluorescence lifetime [23], which provides us with an important scientific reference for the FL enhancement mechanism of CDs (such as the choice of effective dopants). However, the carbon precursor used by Dong is the universally applicable citric acid, and we believe that replacing this reagent with food waste is more economical and environmentally friendly. Given the low weight-gain risk and delicious taste of crayfish, the demand for crayfish is increasing. According to the survey, “crayfish meat accounts for only 30% of total crayfish weight, while about 420,000~434,000 tons of crayfish shell are produced per year as waste” [24], and it causes serious environmental problems. Crayfish shell is rich in protein and chitin, which meets the carbon source requirement of CDs and inspires us to fully develop and utilize crayfish shell waste in an innovative way. According to report, food waste has previously been introduced as a carbon precursor in carbon dots synthesis [13]. Therefore, in order to verify the unique advantages of fluorescent probes prepared from crayfish shells for detecting TAP, we conducted a large number of preliminary experiments to screen food waste. As shown in Table S2, firstly, compared with other food wastes, the fluorescent probes prepared from crayfish shells have better FL intensities and exhibit better fluorescence responses to TAP. Secondly, compared to the probe without dopant, the probe with dopant, especially L-cysteine, showed the best detection effect for TAP. However, as far as we know, there are no reports for preparing N, S-CDs from crayfish shell waste; notably, this environmental-friendly probe has not been practically used to detect TAP in real food and living cells. It is of great significance for turning waste into value-added products and improving the comprehensiveness of TAP testing methods.

**Figure 1:**
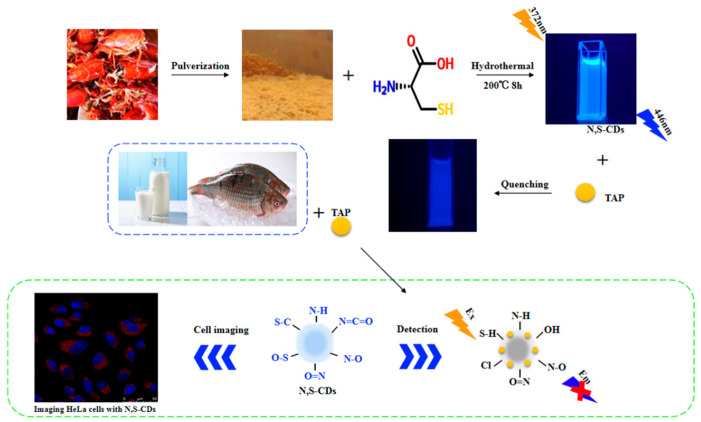
Schematic illustration of the synthesis of N, S-CDs and its application.

Herein, a new method of synthesis based on N, S-CDs is proposed. The readily available crayfish shell waste and L-cysteine were employed as a precursor and dopant for the hydrothermal synthesis of CDs, respectively. The synthesis mechanism diagram is shown in Figure 1. The novel fluorescent materials display a better Abs-QY and stable fluorescent lifetime, and thus, the probe can be effectively quenched by TAP via static quenching. The results demonstrate that the method our proposed can rapidly detect trace TAP in food samples within 45 min without interference from complex environment (including sample pretreatment time). Importantly, the cell viability remained 91.76% at the high concentration of N, S-CDs, and TAP can be also smoothly tracked by N, S-CDs in living cells. It is anticipated that this eco-friendly fluorescence probe may be widely used in monitoring food quality and as a fluorescent-imaging agent in pharmaceutical analysis.

## 2. Materials and Methods

### 2.1. Materials

Crayfish shells were collected from restaurant waste in Changsha, China. The reference pharmaceuticals thiamphenicol (TAP), florfenicol (FF), chloramphenicol (CAP), amoxicillin (AMX), sulfonamide (SDI), penicillin (PG), doxycycline hydrochloride (DOX), melamine (MEL) (≥98%), 2-(4-Amidinophenyl)-6-indolecarbamidine dihydrochloride (DAPI), and dimethyl sulfoxide (DMSO) were provided by Shanghai yuan ye Bio-Technology Co., Ltd. (Shanghai, China). L-cysteine was purchased from Chengdu Kelon Chemical Reagent Co., Ltd. (Chengdu, China). Britton–Robinson (BR) buffer solutions were prepared from H_3_PO_4_–HAc–H_3_BO_3_ mixed-acid solution. All the reagents were of analytical grade and used without further purification. Hela cell lines were obtained from Shanghai Binxin Bio-Technology Co., Ltd. (Shanghai, China). Ultrapure water (18.2 MΩ·cm) was produced by a Milli-Q ultrapure water system (Millipore, Burlington, MA, USA). Milk and fish samples were obtained from the Ya’an Jixuan supermarket (Ya’an, China).

### 2.2. Synthesis of N, S-CDs

N, S-doped CDs were prepared by hydrothermal treatment of crayfish shells. Crayfish shells (2 g) were pulverized and dispersed in 60 mL of ultrapure water, followed by the addition of L-cysteine (1 g). The mixture was transferred to a reaction kettle and then heated in an oven (200 °C, 8 h). After cooling to room temperature, the brownish dispersion was centrifuged at 12,000 rpm for 5 min to remove the precipitate. The supernatant was then collected by filtration (0.22 μm) and dialyzed (MW, 1000 Da) for 2 days to purify the N, S-CDs solution. Lastly, the resulting products were dried by lyophilization (−80 °C) to obtain N, S-CDs powder. Then, it was dispersed in water for further characterization and application.

### 2.3. Characterization of N, S-CDs

The morphologies and structures of N, S-CDs were explored by transmission electron microscopy (TEM) and high-resolution TEM (HRTEM) (JEOL-2100F electron microscope JEOL, Tokyo, Japan). The FTIR spectrum in KBr was acquired on a NICOLEF IS 10 FTIR apparatus (Thermo Fisher Scientific Co., Ltd., Waltham, MA, USA). The phase and elemental analyses were performed on a D/Max-2500 X-ray diffractometer (XRD, Shimadzu, Kyoto, Japan) and X-ray photoelectron spectroscope (XPS, ESCALAB250, Thermo, Waltham, MA, USA). The PL spectra and UV–vis absorption spectra were measured on a Varioskan Flash multimode reader (Thermo Fisher Scientific Co., Ltd., Waltham, MA, USA). Fluorescence lifetime measurements were performed with an Edinburgh FLSP920 time-corrected single photon-counting system. The cell imaging was taken by laser confocal fluorescence microscope (TCS SP5 II, Beijing Zhongyi Optical Technology Development Co., Ltd., Beijing, China). The Abs-QY of N, S-CDs were measured through a calibrated integrating sphere (IS) in the FLSP920 spectrometer (Edinburgh Instruments Ltd., Livingston, Scotland, UK). The Abs-QY value was calculated [25] as (Equation (1)):

(1) $$\eta =\frac{Eb-Ea}{Sa-Sb}$$

where η is the absolute fluorescence quantum yield, the indices “*a*” and “*b*” referred to the reference sample (deionized water) and carbon dots aqueous solution separately, and *Ea*, *Eb*, *Sa*, and *Sb* referred to the integral of the scans.

### 2.4. Sensing Approaches for TAP Determination

The obtained N, S-CDs were mixed with BR buffer solutions (volume ratio: 2:1), and then, different concentrations of TAP (50–300 μg·L^−1^, 20–50 μg·L^−1^) were added into the mixture to form the N, S-CDs-TAP system (volume ratio of N, S-CDs, BR buffer, and TAP: 2:1:2), followed by vortexing the mixture for 30 s. The PL intensities were recorded at a λ_ex_/λ_em_ of 376/446 nm, and a linear standard curve for TAP sensing by N, S-CDs was established by the ratio of the fluorescence intensity of probes in the absence (F_0_) and presence (F) of different concentrations of TAP. The selectivity of the detection system was investigated. Specifically, various representative antibiotics (FF, CAP, AMX, SDI, PG, DOX, and MEL) at the same concentration were added into the system instead of TAP, and the fluorescence intensity ratio were calculated under the same conditions (λ_ex_/λ_em_ of 376/446 nm). Interference experiments were performed by directly adding some biomolecules and metal ions (e.g., Ca^2+^, Mg^2+^, Na^+^, Cl^−^, K^+^, sucrose, glucose, fructose, urea, and L-ascorbic acid) of high concentration (100–5000 mg·L^−1^), seen commonly in the food matrix, to the N, S-CDs-TAP system.

### 2.5. Analysis of Real Food Samples

Herein, the fish and milk real food samples were prepared and tested following a standard procedure to evaluate the eco-friendly probe’s applicability in detecting TAP in foods [26,27]. Briefly, 2 g of spiked fish and milk samples (1 and 2 and 3 times that of the MRL value, respectively) [28] were placed in 50 mL centrifuge tubes, and 20 mL of ethyl acetate was added followed by thorough mixing. After centrifuging at 8000 rpm for 5 min to remove the protein, the extraction process was repeated twice. The supernatant of fish and milk samples were concentrated by nitrogen flow at 40 °C and re-dissolved in 5.0 mL ultrapure water. In the next step, this suspension was filtered through a 0.22 μm membrane filter. BR buffer and N, S-CDs solution were added into the solution and then vortexed for 3 min to obtain the sample ready for analysis. All the experiments were performed in triplicate.

### 2.6. Cellular Toxicity Test

The cytotoxicities of the N, S-CDs were evaluated on Hela cells by using colorimetric MTT assays [29]. Firstly, the Hela cells were cultured in the DMEM medium containing 10% fetal bovine serum to 7.5 × 10^4^ cells·mL^−1^. Then, the as-obtained Hela cells were taken out as aliquot in 96-well plates containing N, S-CDs with different concentrations (0−550 μg·mL^−1^) and incubated for 24 h in an incubator. After 72 h of incubation, the cells were lysed with dimethyl sulfoxide (DMSO), and the absorbance of MTT at 570 nm optical density of 96-well plate was counted with microplate reader (Thermo Fisher Scientific Co., Ltd., Waltham, MA, USA). The cell viabilities were calculated according to following formula (Equation (2)):

(2) $$cell\;viability\%=\left(\frac{OD_{sample}}{OD_{blank}}\right)\times 100\%$$

where *OD_blank_* is obtained in the absence of the sample, and *OD_sample_* is obtained in the presence of N, S-CDs sample.

### 2.7. Cellular Imaging and Intracellular Detection of TAP in Hela Cells

The potential bioimaging application of the N, S-CDs was evaluated against the Hela cells through a procedure reported previously in the literature [14,30]. Firstly, the Hela cells were collected in the logarithmic growth phase and adjusted to 7.5 × 10^4^ cells·mL^−1^ in DMEM medium with fetal bovine serum (10%). Then, they were seeded on 22 × 24 mm with sterile cell slides and incubated overnight at 37 °C under 5% CO_2_. Afterward, discarding the supernatant, the culture medium was replaced with N, S-CDs containing serum-free medium for 24 h. The cells in coverslips were washed three times with PBS buffer, and 4% PFA was fixed for 15 min at 4 °C and washed three times with PBS. Lastly, the cells were stained for 15 min at room temperature using TAP or DAPI, followed by washing three times with PBS. Fluorescence imaging of the cells was observed using a laser confocal microscope. Each variable was assessed in triplicate.

## 3. Results and Discussion

### 3.1. Characterization of N, S-CDs

**Figure 2:**
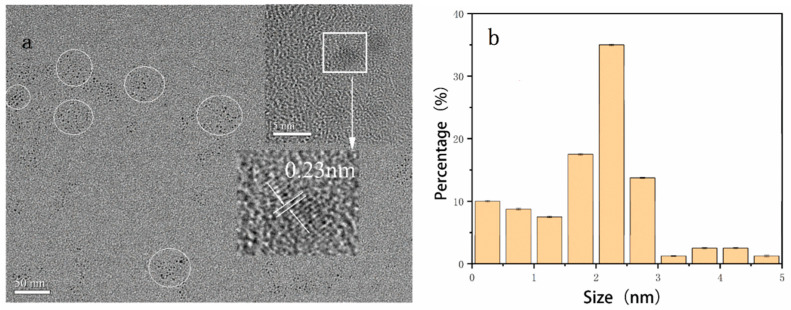

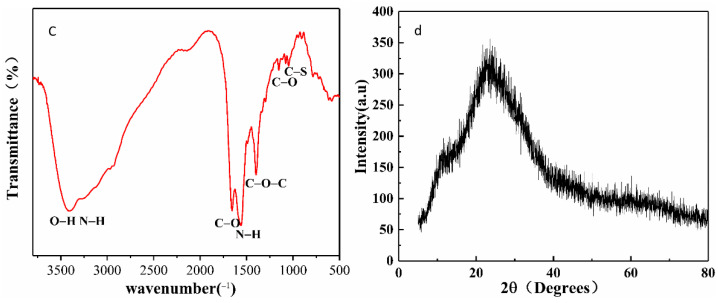
(**a**) The TEM imaging of the N, S-CDs; inset: the HRTEM imaging of the N, S-CDs; (**b**) the size distribution of the N, S-CDs; and (**c**) the FT-IR (**d**) and XRD of the N, S-CDs.

The synthesis mechanism diagram of N, S-CDs is shown in Figure 1. As shown in Figure 2a, the TEM results showed that N, S-CDs were homogeneous in size and morphology, without any obvious aggregation. A clear lattice fringe with a lattice spacing of 0.23 nm is displayed in the inset of Figure 2a, which can be interpreted as the (100) planes diffraction of the graphitic carbon framework [31]. It reveals the easy carbonization of the crayfish shell waste precursors to form a graphitic microstructure within the N, S-CDs. The average particle size diameter of carbon dots were generated by ImageJ software (Figure 2b). It can be observed that the average diameter of N, S-CD obtained in this study is 2.38 ± 0.14 nm.

**Figure 3:**
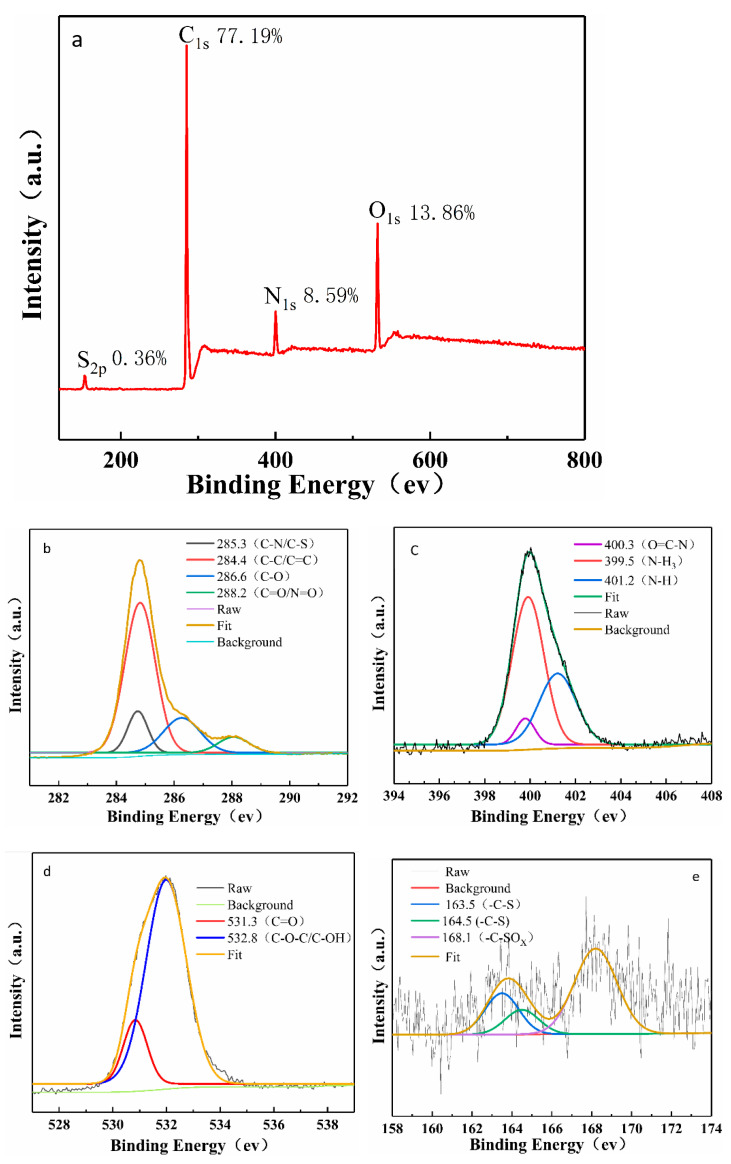
(**a**) The full-scan XPS spectrum of the N, S-CDs and (**b**) the high-resolution XPS of C1s, (**c**) N1s, (**d**) O1s, and (**e**) S2p.

In the spectrum shown in Figure 2c, the broad absorption band in the region of 3000–3404 cm^−1^ can be assigned to O-H and N-H bending vibrations. The characteristic absorption peaks at 1656 and 1562 cm^−1^ could put down to the stretching vibration of C=O and N=O groups, respectively, and the 1397 cm^−1^ peak can be confirmed as C-N and -COO stretching bands [15,32]. Moreover, the C-O and C-S stretching bands [33] were situated at 1050 cm^−1^ and 1153 cm^−1^. The peaks located at 1050 cm^−1^ and 787 cm^−1^ correspond to the C-O stretching vibrations and in-plane bending of the C-O bond [29], respectively. Lastly, a strong N-H stretching vibration [34] was observed at 669 cm^−1^. Consequently, these FTIR spectral data demonstrated the existence of C=O, -NH_2_, -OH, and C-S and C-N functional groups on the surface of N, S-CDs. It is apparent from Figure 2d that the XRD pattern of N, S-CDs displayed a typical wide peak at about 23.6°, which can be interpreted as the (002) planes of the graphitic carbon structure, confirming the amorphous carbon structure of N, S-CDs [21]. This observation may be attributed to the rich nitrogen, sulfur, oxygen, and other functional groups introduced [34] by the N, S-CDs sample. The surface functionalization process of N, S-CDs was further studied by XPS spectra. Four typical peaks at 170.1 eV, 289.9 eV, 400.8 eV, and 535.2 eV, as shown in Figure 3a, can be assigned as corresponding to S2p, C1s, N1s, and O1s, respectively [29]. Figure 3b demonstrated the four states of carbon atoms were C=C/C-C (284.4 eV), C-N/C-S (285.3 eV), C-O (286.6 eV), and C=N/N=O (288.2 eV), respectively [33]. The N1s XPS spectrum displayed three peaks at N-(H)_3_ (399.5 eV), O=C-N (400.3 eV) [18], and N-H (401.2 eV) (Figure 3c). Two peaks located at 531.3 eV and 532.8 eV were observed in the O1s XPS spectrum (Figure 3d), which can be attributed to C=O and C-O-C/C-OH functional groups [32]. The high-resolution spectrum of S2p could be divided into three main peaks (Figure 3e), wherein the peaks at 163.5 eV and 164.5 eV were assigned to S2p3/2 and S2p1/2 of the C-S. The emergence of a peak at 168.1 eV could be attributed to the induction of sulfate or sulfonate (-C-SO_3_^−^) [15]. The above results indicated graphitic nature in carbon dots structure and verified the successful synthesis of N, S-CDs.

### 3.2. Optical Properties of N, S-CDs

**Figure 4:**
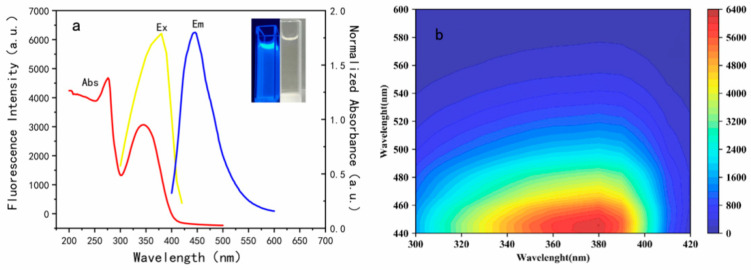
(**a**) Absorption and PL emission spectra (λ_ex_ = 372 nm) of the N, S-CDs; (**b**) PL emission spectra of the N, S-CDs at increasing excitation wavelengths from 300 nm to 420 nm.

According to the Figure 4a, a typical absorption peaks at 265 nm could be interpreted as the π-π* (C=C or C=O) [35] and n-π* (C=N or C-OH) electronic transitions. Studies have shown that doping properly could cause the change of local surface state and the recombination of excited-state energy, promoting the strong fluorescence emission of N, S-CDs in the form of main chromophore, which caused these characteristic absorptions to occur [31,36]. It can be found that the fluorescence intensity of N, S-CDs increased initially and then decreased, increasing the excitation wavelength (Figure 4b), and when excited at 372 nm, the N, S-CDs reflects the strongest blue fluorescence (inset of Figure 4a) near the emission peak of 446 nm. It is noteworthy that the positions of the emission peaks did not change significantly; this phenomenon exhibits that the PL emission of N, S-CDs does not depend on excitation, avoiding the interference of different excitations on the peak position of the sample emission [37], to achieve more accurate detection in complex food samples. Quantum yield is crucial for analyzing the properties of luminescent materials, and different from the relative quantum yield, the absolute quantum yield (Abs-QY) can remove the need for a known luminescence standard as a reference, thus avoiding obvious experimental system errors and accidental errors, and significantly simplifies the measurement process [38,39]. The Abs-QY of CDs and N, S-CDs were calculated to be 18.6% and 50.2%, respectively, which shows that nitrogen-sulfur doping significantly improves the absolute quantum yield of fluorescent probes. Compared with the CDs obtained by green synthesis in other reports, the QY of products in this research increased by two times [14,40]. Additionally, the stability of N, S-CDs in fluorescence and storage was also evaluated.

### 3.3. Stability of the N, S-CDs

**Figure 5:**
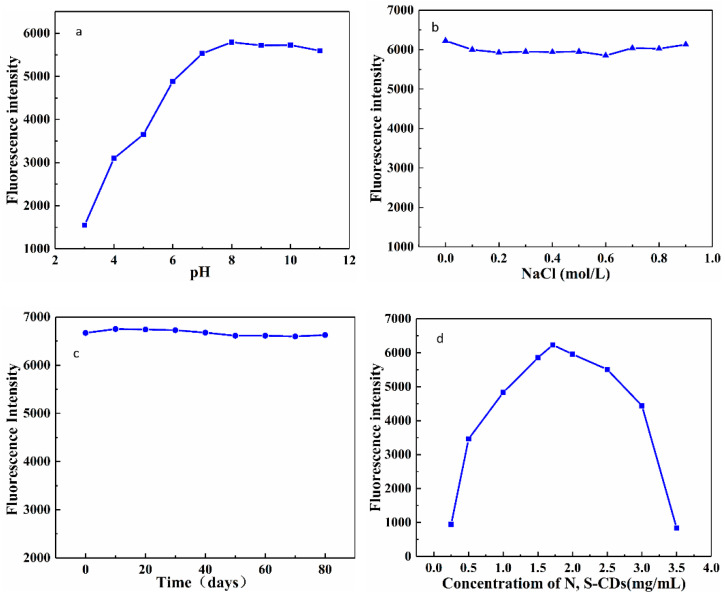
(**a**) Effect of pH on the PL intensities of the N, S-CDs; (**b**) effect of NaCl concentration on the PL intensities of the N, S-CDs; (**c**) effect of storage time on the PL intensities of the N, S-CDs; (**d**) effect of N, S-CDs concentration on the PL intensities of the N, S-CDs.

Figure 5a exhibits the emission behavior of N, S-CDs at different pH values (pH = 3–11). The PL intensity increased initially with an increase in the pH and then remained fairly constant in alkaline conditions. It shows a maximum value at pH = 8 (Ex = 372 nm, Em = 446 nm), which is believed to due to the presence of protonation and deprotonation. As shown in Figure 5b, the N, S-CDs possess excellent photoluminescence stability in the solutions at different ionic strengths (according to the concentration of NaCl), indicating that the N, S-CDs have high salt tolerance. At the same time, it could be observed that there is no substantial alteration in the fluorescence intensity with the storage time in 80 days (Figure 5c), which demonstrates that N, S-CDs have excellent stability for practical applications. Moreover, the proper concentration of N, S-CDs is crucial to the sensitivity of the fluorescent probe. According to reports, excessively high concentration of quantum dots does cause poor dispersion in aqueous solutions due to the generation of numerous nanoparticle clusters, and its non-radioactive energy transfer will also reduce the sensitivity of the method [41]. Therefore, the concentration of N, S-CDs in the range of 0.25–3.5 mg·mL^−1^ was also investigated (Figure 5d). With the increase in the concentration of N, S-CDs, the fluorescence signal showed an initial trend of rising and then falling and reached the maximum fluorescence intensity at 1.75 mg·mL^−1^.

### 3.4. Optimization of the Sensing Conditions

**Figure 6:**
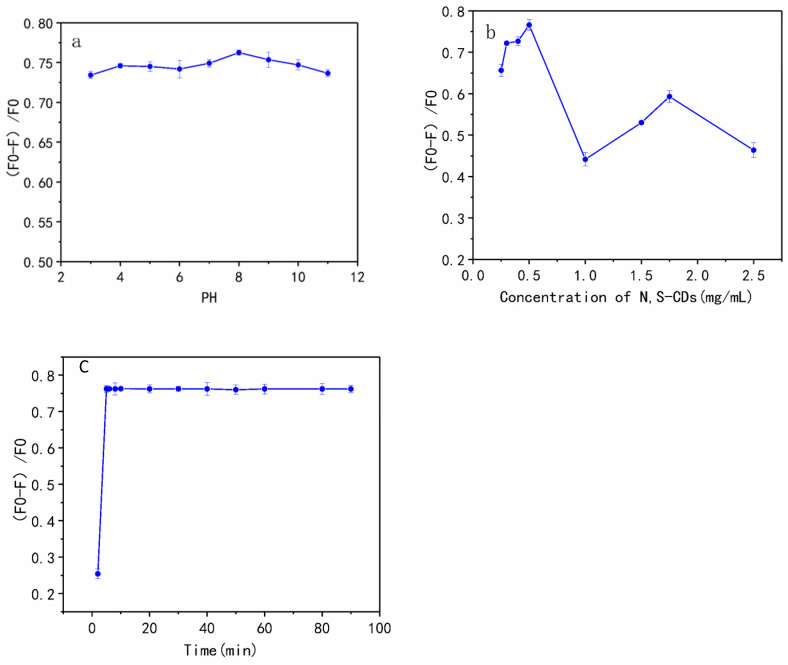
(**a**) The effect of pH on the fluorescent intensity of N, S-CDs-TAP system; (**b**) the effect of N, S-CDs concentration on the fluorescent intensity of N, S-CDs-TAP system; (**c**) the effect of incubation time on the fluorescence intensity of N, S-CDs.

pH has a significant influence on the fluorescence intensity of carbon dots and their interactions with target analytes. Figure 6a depicts the change in the quenching efficiency (F_0_ − F)/F_0_ of fluorescent probes on interaction with TAP in the pH range of 3–11. The PL intensities of N, S-CDs in the absence and presence of TAP are denoted by F_0_ and F, respectively. The fluorescence intensity ratio (F_0_ − F)/F_0_ was the strongest at pH = 8, but at the same time, it remained nearly constant over a wide pH range. Therefore, the detection system is not restricted by different acid-base environments, which is highly advantageous for the application of probes to the food testing and biomedical industries. Since the probe shows a larger quenching efficiency at pH 8 than at other pH ranges, the BR buffer with a pH value of 8 was chosen as the solvent for the detection of TAP. As shown in Figure 6b, the effect of N, S-CDs concentration on quenching efficiency was also investigated. With the increase in the concentration of N, S-CDs, (F_0_ − F)/F_0_ shows an upward trend, reaching the maximum at a concentration of 0.5 mg·mL^−1^, and the continuous increase in the probe concentration leads to an unstable trend where the ratio of quenching repeatedly decreases and increases. As mentioned above, the excessive concentration of N, S-CDs produces too many nanoparticle clusters, which not only reduces the dispersibility of the CDs in the solution but also is not conducive to full interaction with the target. As such, 0.5 mg·mL^−1^ was chosen as the optimal concentration of N, S-CDs for the detection system. It is obvious from Figure 6c that after adding N, S-CDs to the TAP solution, the interaction of TAP with N, S-CDs occurred instantaneously. The fluorescence intensity ratio of the system reached an equilibrium within 5 min and was stable for at least 1.5 h, displaying the advantage of the probe for rapid detection. According to the analysis of optimization experiments, the reaction time, pH, and concentration of N, S-CDs, were determined to be 5 min, pH 8, and 0.5 mg·mL^−1^, respectively, as the conditions for subsequent experimental analysis.

### 3.5. Sensing of N, S-CDs to TAP

**Figure 7:**
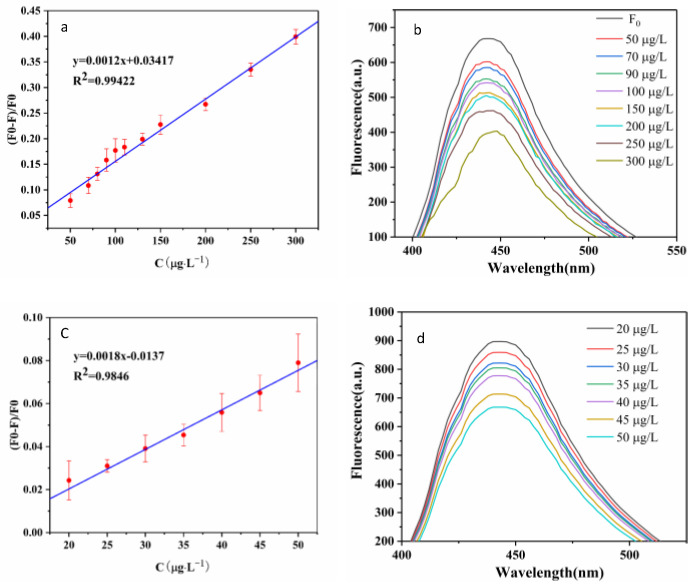
(**a**) The relationship between the fluorescence intensity and different concentrations of TAP (50–300 μg·L^−1^); (**b**) fluorescence spectra of N, S-CDs after adding TAP (50–300 μg·L^−1^); (**c**) the relationship between the fluorescence intensity and different concentrations of TAP (20–50 μg·L^−1^); (**d**) fluorescence spectra of N, S-CDs after adding TAP (20–50 μg·L^−1^).

Under optimized conditions, this fluorescence probe was evaluated for TAP detection. As illustrated in Figure 7a,b, FL intensity of N, S-CDs significantly weakened as TAP concentration rose at the excitation wavelength of 372 nm, which showed good linearity at TAP concentrations from 50–300 μg·L^−1^, with a linear equation of (F_0_ − F)/F_0_ = 0.0012CTAP + 0.03417 (R^2^ = 0.9942), where F_0_ and F are the fluorescence intensities of the detection system without and with TAP, respectively. Moreover, the limit of detection (LOD) was confirmed as 27.5 μg·L^−1^, calculated according to the formula LOD = 3σ/S. As shown in Figure 7c,d, a linearity at TAP concentrations from 20–50 μg·L^−1^, with a linear equation of (F_0_ − F)/F_0_ = 0.0018CTAP − 0.0137 (R^2^ = 0.9846), the LOD was calculated as 11.12 μg·L^−1^. All data indicate that the developed method can quantitatively detect the TAP and could achieve satisfactory results, such as detection limits below national standards.

### 3.6. Selectivity of N, S-CDs to TAP

The selectivity and interference of the N, S-CDs were evaluated to confirm the applicability of fluorescence sensors for food inspection. Several biological agents that are easily residual in food matrices were used to assess the selectivity of N, S-CDs. As shown in Figure S1, among these biological molecules, nonstructural analogues of thiamphenicol did not exhibit significant response effects (amoxicillin, sulfonamide, doxycycline, hydrochloride, melamine). However, chloramphenicol-based antibiotics, such as thiamphenicol, florfenicol, and chloramphenicol, exerted a significant response, and TAP had the highest fluorescence quenching efficiency. Afterward, we performed tests to verify the possibility of biological molecules that conventionally exist in milk samples interfering with the detection system by conducting interference test. Table S3 shows the investigated interference of the detection system. Little changes were observed in the FL intensity of N, S-CDs-TAP system under various interference factors such as high concentration 100–5000 mg·L^−1^. The relative error was less than 2%.

### 3.7. Sensing Mechanism of N, S-CDs to TAP

To clearly understand the sensing mechanism of TAP detection, a series of researches were discussed, including zeta potential and variable temperature experimental (Ster-Volmer equation) analysis, UV–vis absorption spectrometry, and fluorescence lifetime analysis. The quenching process comprises energy transfer, ground-state complex formation, and collisions, etc. [34]. As shown by Figure S2A, compared to the absorption spectrum of N, S-CDs alone (curve b), the absorbance at 280 nm increased significantly after the addition of TAP quencher (curve a); moreover, a weak absorption peak appeared at 300 nm, which was attributed to the additional peak of the newly formed ground-state complex between the fluorophore and the quench agent through π–π* stacking. Besides, the UV–vis spectrum of TAP exhibited a wide absorption peak (curve c) at around 220–300 nm. There was no overlap between N, S-CDs emission and TAP absorption; thus, inner filter effect (IFE) cannot be explained as the quenching mechanism of the detection process [29,42]. There should have been some interactions between N, S-CDs probes and TAP molecules. It is assumed that hydroxyl and halogen atoms of TAP form ground-state complexes such as amino and carboxyl groups or sulfhydryl groups with nitrogen-hydrogen bonds and carbonyl groups of N, S-CDs, which caused aggregation and hence reduced the π-π* excitation in the N, S-CDs, finally resulting in the quenching of the fluorescence. Generally, the understanding of luminescence (fluorescence) decay dynamics of excited states of carbon dots is significant for understanding its optical mechanism and catalytic properties [43]. The fluorescent lifetime can represent the information about the excited state of matter and the presence of a dynamic quencher to shorten the fluorescence lifetime of an excited molecule fluorophore. In contrast, the static quencher does not apparently change the fluorescence lifetime of a fluorophore [42]. The fluorescence decay curves of N, S-CDs with and without TAP were measured, as depicted in Figure S2B. The fluorescent lifetime of N, S-CDs was determined to be 1.72 ns, which turned out to be 1.58 ns after the addition of TAP. The almost constant values demonstrate that the interaction between TAP and N, S-CDs belongs to the category of a static quenching process. Dynamic quenching depends upon diffusion. When the temperature rises, the viscosity of the solution decreases and the movement of molecules accelerates the results in larger diffusion coefficients, and then, the bimolecular quenching constants are supposed to increase. On the contrary, the increase in temperature may cause the stability of the complex to decrease, thereby reducing the degree of static quenching. Comparing the Stern–Volmer quenching constant (KSV) according to the Stern–Volmer equation, the slope obtained for the data recorded at high temperature is lower than that at low temperature (Table S4), which suggests that the mechanism of TAP-induced quenching of N, S-CDs excludes the possibility of dynamic collisions but was attributed to the formation of complexes [11]. The zeta potential of N, S-CDs was measured to be nearly −17.4 mV, which was attributed to the large number of nitrogen hydrogen bond and carbonyl groups present on the surface of N, S-CDs. However, the zeta-potential dropped to −26.70 mV after adding TAP to the N, S-CDs solutions, showing that the hydroxyl and hydrogen bonds in TAP interact with N, S-CDs to generate complexes such as carboxyl and amine groups, which is further validation of the static quenching results [36].

### 3.8. Detection of Actual Food Samples

To further verify this probe’s feasibility and applicability, the proposed method was applied to test TAP concentration in some real food samples such as milk and fish. The results are presented in Table 1.

As Table 1 shows, the obtained recoveries of TAP from spiked milk and fish samples ranged from 97.30 to 99.34%, with the relative standard deviation (RSD) values all being below 2% (*n* = 3). The data reveal that the environment-friendly fluorescent nanoprobe based on N, S-CDs proposed in this research could be a practical approach for accurate TAP detection in complex food. The analytical characteristics of the other reported methods are listed in Table 2. Compared with ELISA techniques, the probes developed in this work do not require using susceptible and highly expensive bio-recognition molecules (such as antibodies, aptamers, or enzymes) and has a comparable linear range (50–300/20–50 μg·L^−1^). Furthermore, the LOD (27.5/11.12 μg·L^−1^) values obtained by the present method are better than UV–vis spectrophotometric methods. Although better detection limits were achieved with chromatographic and electrochemical methods, these require elaborate and sophisticated instrumentation and labor-intensive operation. Importantly, as for the method our developed, the fast and effective pretreatment process can effectively shorten the time to detect TAP in real foods (45 min). Meanwhile, compared with other CDs fluorescent probes [31,35], this probe has faster fluorescence response time (5 min) and benefits from its excellent fluorescence properties such as high quantum yield and stable fluorescence lifetime.

**Table 2:** Comparison of different methods for the detection of TAP.

Technique	Pretreatments	Detection Time	Linear Range (μg·L^−1^)	LOD (μg·L^−1^)	Recovery (%)	Ref.
ELISA	Prepare and screen the polyclonal antibodies	>70 min	0.41–11.2	0.15	77.2–116.0	[7]
UV spectrophotometric	Purification	55 min	5000–25,000	590	99.26–100.56	[8]
HPLC	Thin-layer chromatography (TLC) purification	>1 h	50–10,000	20	82.0–114.9	[9]
HPLC-DAD	dispersive solid phase extraction (SPE)	2 h	5–150	0.48	81.7–97.5	[44]
UHPLC-MS	SPE	>1 h	1–200	0.5	87.6–102.7	[1]
HPLC-ESI/MS	Accelerated solvent extraction (ASE)	>1 h	1–150	0.4	88.3–107	[10]
Fe3O4 @ SiO2-MIPS	Purification and separation by organic solvents	90 min	100–300	10	92.6–95.6	[5]
CNTs-AuNPs electrochemical sensor	Modification of screen printed electrode	>30 min	35.5–10,650	1.07	94.0–97.0	[45]
N, S-CDs	Purification	45 min	50–300	27.5	97.30–99.34	This work

### 3.9. Cytotoxicity and Cellular Imaging

**Figure 8:**
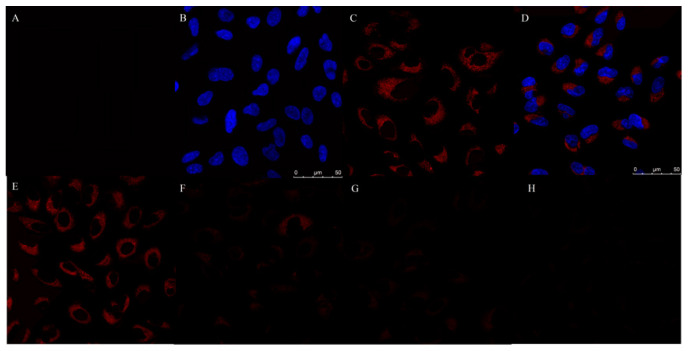
The confocal fluorescence images of Hela cells incubated with N, S-CDs or DAPI; (**A**) control group; (**B**) DAPI; (**C**) N, S-CDs; (**D**) overlap of N, S-CDs, and DAPI; (**E**) the confocal fluorescence images of Hela cells labeled with N, S-CDs (control group); (**F**) confocal fluorescence images of Hela cells labeled with N, S-CDs and TAP (50 μg·mL^−1^); (**G**) confocal fluorescence images of Hela cells labeled with N, S-CDs, and TAP (100 μg·mL^−1^); and (**H**) confocal fluorescence images of Hela cells labeled with N, S-CDs and TAP (250 μg·mL^−1^).

Figure S3 exhibits the extremely low cytotoxicity of N, S-CDs, and the cell viability of Hela cells can still reach 91.76% even at the incubation of 550 μg·mL^−1^ of N, S-CDs. Because of the low toxicity assay and outstanding optical merits of N, S-CDs mentioned above, this probe was used as a means for the fluorometric detection of TAP in living Hela cells. The cells were treated previously with N, S-CDs. It is clear from Figure 6A–D that the non-treated cells showed almost no background fluorescence, while the cells with N, S-CDs showed a light fluorescence; the results reveal that N, S-CDs can penetrate the cell membrane without restriction and be positioned in the cytoplasm. At the same time, it makes the intracellular biosensing feasible. From Figure 8E–H, it is noticeable that compared to the control cells, those supplemented with TAP (50, 100, 250 μg·mL^−1^) have a significant decrease in fluorescence intensity. This highlights the advantages and great potential of N, S-CDs as a novel bioluminescent probe for monitoring TAP in living cells.

## 4. Conclusions

In summary, a novel, facile, and low-cost fluorescent probe N, S-CDs prepared from crayfish shell waste precursors was utilized for the selective detection of TAP and its analogues. The probe exhibited high fluorescence activity with Abs-QY of 50.2% and stable fluorescence lifetime and displayed outstanding properties in terms of detecting the TAP in food samples, for example, wide linear range (20–300 μg·L^−1^), high selectivity, and good limits of detection (LOD = 11.12 μg·L^−1^). Specifically, the rapid detection time was within 45 min compared with other methods and the recoveries varied from 97.30% to 99.34% with the RSDs < 2%. We concluded that the formation of a non-fluorescent complex from N, S-CDs-TAP system is most likely accountable for the quenching phenomenon. Furthermore, the results proved that the probes made of environmentally friendly materials exhibited significantly low toxicity and FL intensity, which further demonstrated the potential of intracellular detection of TAP and cell imaging. More effort should be devoted to the commercialization of the N, S-CDs due to their low cost, environmentally friendly nature, and simple and rapid detection method, which provides new insights into food quality monitoring and biomedical control fields.

## 5. Patents

A patent for invention resulted from the work reported in this manuscript: A fluorescent probe of carbon quantum dots and a method for detecting the content of thiamphenicol (Patent number: ZL 2021 1 0156639.9).

## Figures and Tables

**Table 1:** Determination of TAP in spiked real samples by N, S-CDs (*n* = 3).

Sample	Added (μg·L^−1^)	Found (μg·L^−1^)	Recovery	RSD (%)
Milk-1	0	nd ^a^	-	-
Milk-2	50	49.20 ± 0.77	98.39%	1.54%
Milk-3	100	98.20 ± 1.04	98.20%	1.04%
Milk-4	150	149.00 ± 1.33	99.34%	0.89%
Fish-1	0	nd ^a^	-	-
Fish-2	50	48.91 ± 0.20	97.30%	0.41%
Fish-3	100	96.16 ± 0.67	97.86%	0.69%
Fish-4	150	148.64 ± 1.99	97.92%	1.34%

nd ^a^ defined as “not detected”.

## Data Availability

Data are contained within the article.

## Supplementary Materials

The following supporting information can be downloaded at: https://www.mdpi.com/article/10.3390/foods11162414/s1, Figure S1: selectivity of the N, S-CDs to different kinds of antibiotics; Figure S2: Supplementary experimental content: Sensing mechanism of N, S-CDs to TAP; Figure S3: Cytotoxicity of the N, S-CDs toward Hela cells; Table S1: Comparison of N, S-CDs prepared from different precursor; Table S2: Comparison of fluorescence response of N, S-CDs prepared from different food wastes and dopant to TAP; Table S3: Effect of interfering substances of C-dots-TAP solution system; Table S4: Comparison temperatures of different research for the calculated KSV values; Table S5. Specification information for N, S-CDs. References [46,47,48] are cited in the supplementary materials.
