# Supplementary material for: Facile Synthesis of N, S-Doped Carbon Quantum Dots from Food Waste as Fluorescent Probe for Sensitive Detection of Thiamphenicol and Its Analogues in Real Food Samples along with an Application in Bioimaging
Source: Foods. 2022 Aug 11;11(16):2414. doi: 10.3390/foods11162414 (PMC9407342; doi:10.3390/foods11162414)
Supplement: Supplementary file 1 [file foods-11-02414-s001.zip › foods-1823442-supplementary.pdf]

Supplementary Material

Facile synthesis of N, S-doped carbon quantum dots from food waste as fluorescent probe for sensitive detection of thiamphenicol and its analogues in real food samples along with an application in bioimaging

Shujuan Chen [a, 1,*], Wanlin Ouyang [a,b,1], Jianlin Zhang [a], Li He [a], Likou Zou [c], Xiaolin Ao [a], Shuliang Liu [a], Yong Yang [a], Jianlong Li [a]

[a] College of Food Science, Sichuan Agricultural University, Ya'an, Sichuan 625014, PR China

[b] Yi'yang Agricultural Products Quality Inspection and Testing Center, Yi'yang, Hunan 413000, PR China

[c] College of Resources, Sichuan Agricultural University, Chengdu, Sichuan 611130, PR China

[1] The authors contributed equally to this work.

***Correspondence: chenshujuan1@163.com (Shujuan Chen).**

Table S1. Comparison of N, S-CDs prepared from different precursor.

Precursor	Quantum Yield	Cell viability	Detection objects	Samples	Ref.
L-cysteine	12.6%	Not mentioned	Hg^{2+}	Lake water sample	[16]
Citrate	16.1%	85%	Hg^{2+}	Tap/drinking water	[17]
$C_3N_3S_3$	30.4%	80%	TC	Living cells	[18]
Thiosemicarbazide	12%	Not mentioned	Cr^{6+}	distilled water	[19]
Cystamine dihydrochloride	39.7%	78 %	Cr^{6+}	deionized water	[20]
1，6-hexanediamine dihydrochloride	24%	81 %	Mn^{7+}	DI water	[21]
Citric acid	17.6%	Not mentioned	Hg^{2+}	Circumstance water	[22]
P. oceanica leaf、ethylenediamine and cysteamine	10.6%	Not mentioned	Fe^{3+}、$Cr_2O_7^{2-}$、CrO_4^{2-}.	water	[46]
Rice and N-acetyl-L-cysteine	7.37%	Not mentioned	-	-	[47]
Frying oil and concentrated sulfuric acid	3.66%	90%	-	-	[48]
N, S-CDs	50.2%	92%	TAP	Milk、fish and living cells	Our work

Table S2. Comparison of fluorescence response of N, S-CDs prepared from different food wastes and dopant to TAP.

Carbon precursor	N, S Dopant	FL intensity (F_0)	After TAP (F)	$(F_0-F)/F_0$
Pitaya peel	-	215.4	201.9	0.063
	Thiamine hydrochloride	382.6	365.1	0.045
	L-cysteine	575.7	427.69	0.25
	Thioacetamide	581.64	482.33	0.17
Grapefruit peel	-	821.6	792.7	0.035
	Thiamine hydrochloride	1342.8	1160	0.14
	L-cysteine	1187.7	1289.2	-0.085
	Thioacetamide	1594.3	896.6	0.44
Bamboo shell	-	184.6	190.9	-0.03
	Thiamine hydrochloride	318.9	355.2	-0.11
	L-cysteine	385.2	390	-0.012
	Thioacetamide	403.1	426.7	-0.059
Fish scales	-	1225.5	878.1	0.28
	Thiamine hydrochloride	2412	2090.3	0.13
	L-cysteine	2033.5	1553.2	0.24
	Thioacetamide	1846.5	1417.8	0.23
Crayfish shell	-	3549.6	2268.1	0.36
	Thiamine hydrochloride	5711	3100.8	0.46
	L-cysteine	6286.2	2547.9	0.71
	Thioacetamide	5102.4	4062	0.40
Chicken feather	-	1066.7	1084.3	-0.016
	Thiamine hydrochloride	1147.4	1189.7	-0.037
	L-cysteine	1169.1	1213.8	-0.038
	Thioacetamide	1289.8	1459.7	-0.13

Table S3. Effect of interfering substances of C-dots-TAP solution system.

Interfering substances	Concentration ($mg \cdot L^{-1}$)	Relative error (%)	Interfering substances	Concentration ($mg \cdot L^{-1}$)	Relative error (%)
Na^+	5000	+0.65	Sucrose	500	+0.35
Cl^-	5000	+1.69	Glucose	500	+1.39
K^+	5000	+0.78	Fructose	500	+0.38
Mg^{2+}	1000	-0.23	Urea	500	+0.67
Ca^{2+}	1000	+0.98	L-ascorbic acid	100	+1.27

Notes: C_{TAP}=100 μg·L^{-1}, n=6.

Table S4. Comparison of different research temperatures for the calculated KSV values.

Temperature（±1K）	288.15	303.15	318
Ksv（$*10^{-3}$）	1.35	1.2	1.15

Table S5. Specification information for N, S-CDs.

Sample	Information		
N, S-CDs	Synthetic substance	Crayfish shell	L-cysteine
	Concentration	1.75 mg·mL^{-1}	
	pH	8	
	Fluorescence stability	80 days	
	Fluorescence lifetime	1.72 ns	
	Ex/Em	Ex: 372nm	Em: 446nm

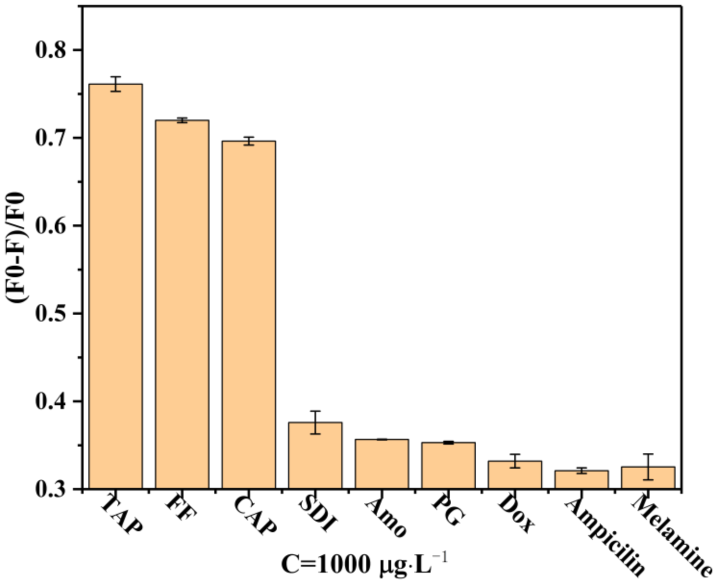

Figure S1. selectivity of the N, S-CDs to different kinds of antibiotics.

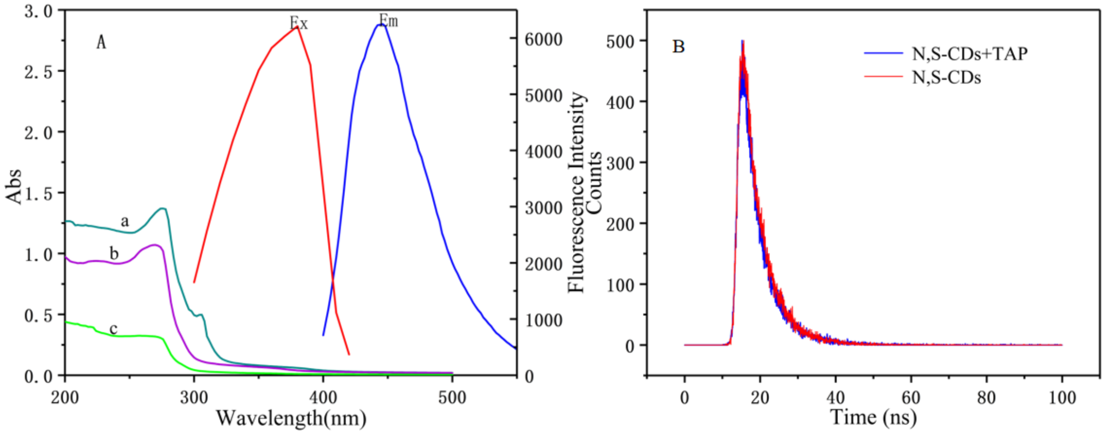

Figure S2. (A) N, S-CDs in the presence (a) and absence (b) of TAP, and (c) Absorption spectra of the TAP; (B) Fluorescence decay curves of pure N, S-CDs and N, S-CDs after interacting with TAP.

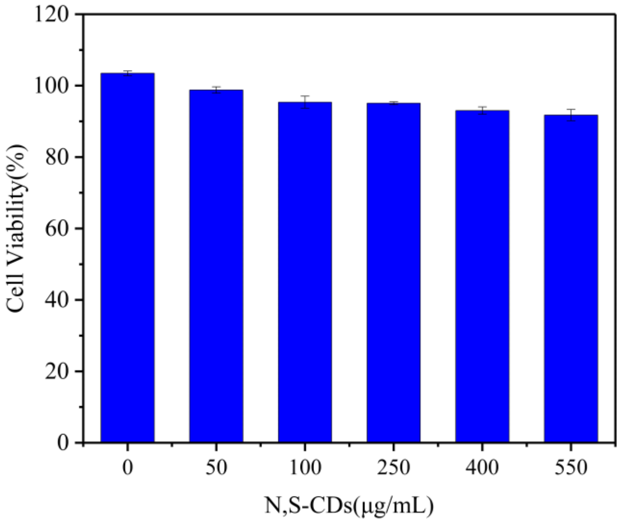

Figure. S3. Cytotoxicity of the N, S-CDs toward Hela cells.
